# Targeting fatty acid metabolism in glioblastoma

**DOI:** 10.1172/JCI163448

**Published:** 2023-01-03

**Authors:** Jason Miska, Navdeep S. Chandel

**Affiliations:** 1 Department of Neurological Surgery and; 2 Department of Medicine, Feinberg School of Medicine, Northwestern University, Chicago, Illinois, USA.

## Abstract

Glioblastoma (GBM) is a primary tumor of the brain defined by its uniform lethality and resistance to conventional therapies. There have been considerable efforts to untangle the metabolic underpinnings of this disease to find novel therapeutic avenues for treatment. An emerging focus in this field is fatty acid (FA) metabolism, which is critical for numerous diverse biological processes involved in GBM pathogenesis. These processes can be classified into four broad fates: anabolism, catabolism, regulation of ferroptosis, and the generation of signaling molecules. Each fate provides a unique perspective by which we can inspect GBM biology and gives us a road map to understanding this complicated field. This Review discusses the basic, translational, and clinical insights into each of these fates to provide a contemporary understanding of FA biology in GBM. It is clear, based on the literature, that there are far more questions than answers in the field of FA metabolism in GBM, and substantial efforts should be made to untangle these complex processes in this intractable disease.

## Introduction

Fatty acids (FAs) are small carbon-rich molecules with diverse functions ranging from lipid bilayer components to signaling molecules used for intercellular communication as well as FA oxidation to generate ATP. In the central nervous system (CNS), FAs are of particular importance, as lipids represent 50% to 60% of the total brain’s dry weight (1). FAs are needed for many CNS processes, including myelin sheath generation (2), axonal growth/regeneration (3), and neurotransmitter trafficking (reviewed in ref. 4). Furthermore, despite glucose being the most envisaged energy substrate in the brain, FAs are readily catabolized in the CNS to generate ATP (5). Highlighting their importance to the CNS, dysregulation of FA metabolism is linked to severe CNS pathologies such as Tay-Sachs disease (6), Gaucher’s disease (7), Alzheimer’s disease (8), Parkinson’s disease (9), and other CNS maladies (10). Despite the vital role of FA metabolism in CNS health, only recently has research begun to understand the role of FA metabolism in tumors that arise from, or metastasize to, the CNS. The results of these studies indicate that targeting of FA metabolic pathways may be an efficacious route to treatment of these malignancies. In this Review, we explore lipid metabolic pathway studies relevant to glioblastoma (GBM) and highlight potential therapeutic avenues of research.

## FA metabolism in the CNS

### Classes of lipids

FAs can be transported around the body either “free” or “bound” to lipoprotein-rich carriers called apolipoproteins. Free fatty acids (FFAs) are not bound directly to a carrier but are instead found circulating attached to albumin (11). Inside the cell, these FFAs are transported or modified by various FA-binding proteins (FABPs) and can be stored in specialized structures known as lipid droplets (12), be used to generate ATP via fatty acid oxidation (FAO) (13), or be used for the generation of new lipid species in the cell (14). Alternatively, lipids may also be transported via lipoprotein carriers called low-density and high-density lipoproteins. These FA “vehicles” transport FAs (usually as triglycerides) and cholesterol around the body from the liver but are minimally transported into the CNS (15). Instead, apolipoprotein E–containing particles are synthesized within the CNS by astrocytes and are the major source of both lipid and cholesterol intercellular transport (16–18). The FAs liberated from these particles can be used in the same processes described for FFAs. There are seven other classes of lipid compounds (19), of which five are pertinent to mammalian CNS physiology: sphingolipids (e.g., sphingomyelin, which is used to make the myelin sheath; ref. 20), glycerophospholipids (which principally make up the lipid bilayer; ref. 21), sterols (cholesterol generated by astrocytes is the best-known example in the CNS; ref. 22), glycerolipid (e.g., triglycerides), and prenols. While these classes of lipid conjugates are critical to both CNS and brain tumor biology, this Review will focus mostly on FAs and their fates within cells.

### Degrees of saturation

FAs have three “levels” of saturation. “Saturated” indicates no double bond formation, “monounsaturated” indicates a single double bond in the FA carbon chain (termed MUFAs), and “polyunsaturated” indicates multiple double bonds in the FA carbon chain (i.e., PUFAs). Saturated FAs and MUFAs can be synthesized de novo in the CNS (23), whereas PUFAs cannot be synthesized de novo without initial dietary uptake (24). The PUFAs α-linolenic acid and linolenic acid must be initially obtained from the diet, and are thus considered “essential” fatty acids (25). These can then be subsequently modified by the cellular machinery. PUFAs are categorized by the distance of the first double bond from the terminal methyl groups, i.e., a double bond on a third carbon is called an omega-3, whereas the first double bond being six carbons away is called an omega-6. The omega-6 FAs linoleic acid and arachidonic acid and the omega-3 FA docosahexaenoic acid are the most abundant PUFAs in the human CNS (24). Like PUFAs, specific saturated and monounsaturated FAs are also found in high quantities in the CNS. The major FA species found in the normal CNS include palmitate and stearate (saturated) and palmitoleic and stearoleic acids (monounsaturated). However, it cannot be overstated how much the lipidomic representation of the brain varies, by age, location, and cell type (26). The de novo synthesis or modification of essential FAs can be performed by many cells in the CNS, with different subsets responsible for producing different lipid species.

## The four categories of FA metabolism in GBM

There are four major metabolic fates of FAs in tumors, and we will discuss each in the context of brain tumor biology. The first is the use of FAs for anabolic processes, or, put simply, building things. In a proliferating cancer cell that is dividing, all lipid metabolic processes will be geared toward making new lipid bilayers, vacuolar membranes, and other biomass processes. The second process is catabolism of FAs to generate energy for the cell. This is most directly associated with α and β fatty acid oxidation (FAO) conducted by peroxisomes and mitochondria, respectively, to produce ATP in a cell. Importantly, anabolic and catabolic processes do not, and cannot, occur simultaneously in a cell, as by-products of biosynthetic/oxidative reactions act as inhibitors for their opposing reactions (13). However, FA anabolism and metabolism may occur simultaneously at different spatial locations within a tumor. For example, the leading edge of a tumor encountering nutrient-replete conditions may be synthesizing new lipids to invade new CNS tissue, while tumor cells near hypoxic, nutrient-depleted regions are consuming lipids to generate ATP for survival. The third fate of FAs is tied to a form of non-apoptotic cell death called ferroptosis, which is regulated by iron, reactive oxygen species (ROS), and PUFA levels in cells. The fourth fate of FAs is the production of signaling molecules. FAs are the precursors to dozens of signaling molecules, with broad effects on both local and systemic physiology.

### FA anabolism in GBM

Fatty acid synthesis (FAS), unlike oxidation, occurs in the cytoplasm, in which citrate acts as a substrate for ATP-citrate lyase (which has been previously targeted using several inhibitors; refs. 27–29) to produce cytosolic acetyl-CoA (13). This occurs in the cytoplasm due to the availability of NADPH, which is a required cofactor for FA biosynthetic reactions. Acetyl-CoA is then used as a building block for the cyclical process that generates palmitate or other even-numbered saturated FAs. The rate-limiting step of FAS is the generation of malonyl-CoA from acetyl-CoA by the acetyl-CoA carboxylase enzymes (ACC1 and ACC2). Malonyl-CoA is then used as a substrate for fatty acid synthase (FASN), a unique multifunctional protein with multiple enzymatic domains that catalyze four subsequent reactions to produce palmitate (10). Every step of these reactions has been implicated in GBM and has been targeted using genetic and pharmacologic approaches.

FAS is principally regulated by the transcription factor sterol regulatory element–binding protein-1 (SREBP-1). This essential growth pathway is governed at multiple levels, and each plays an essential role in GBM pathogenesis. A previous study demonstrated that EGFR signaling resulted in the transcription of lipogenic genes via SREBP-1 activation, and sensitivity to anti-lipogenic therapies was directly related to the amount of tumoral EGFR activity (30). Subsequent work identified that, mechanistically, EGFR signaling induces glucose uptake, which is then used to glycosylate the enzyme SCAP. This glycosylated SCAP can then go to the Golgi apparatus, which is essential in cleaving/activating SREBP-1. Adding another layer of regulation to this pathway, recent work from this same group discovered that the metabolite ammonia (derived from glutamine) plays a critical role in SCAP/Insig dissociation, freeing up SCAP to travel to the Golgi and cleave SREBP-1 (31). This provides another layer of metabolic control of FAS in GBM. However, this is a vast oversimplification of these pathways, and a more detailed review of SREBP-1 and FAS in GBM can be found in refs. 32 and 33.

Since the above studies demonstrate that EGFR signaling is central to FAS in GBM, it is no surprise that GBM with the EGFR variant III amplification (EGFRvIII^+^, the most common EGFR gain-of-function mutation in GBM) has a higher proliferative rate owing to increased de novo lipogenesis and overall metabolic activity (34, 35). Jones et al. (34) found that the use of siRNA against ACC1 and ACC2 resulted in significant inhibition of proliferation and induction of apoptosis in EGFRvIII^+^ cell lines as compared with EGFRvIII^–^ cell lines. In another study examining ACC activity in GBM, the authors discovered that the antifungal sulconazole inhibits biotinylation of ACC1/2 (and other carboxylases) in glioma stem cells. Treatment with sulconazole has potent anti–glioma stem cell activities and results in cholesterol depletion and a compensatory increase in glycolytic phenotypes (36). Interestingly, the results of a recent phase II clinical trial indicated that phospho-ACC can be used as a biomarker for the efficacy of the anti-angiogenic compound regorafenib (37). This finding follows previous research indicating that regorafenib can activate AMPK, which can phosphorylate, and thus inactivate, ACC activity (38). Supporting this hypothesis, previous work identified that agonism of AMPK by AICAR prevents EGFRvIII^+^ GBM cells from growing both in vitro and in vivo (35). Mechanistically these authors also found that AMPK activation–mediated tumor inhibition was regulated by the inhibition of FA biosynthesis. Therefore, the results of these studies indicate that inhibition of the initial steps in FAS may be a useful target of intervention in the treatment of brain tumors.

Several studies have explored the perturbation of the downstream steps of FAS in GBM. In the first study to examine the targeting of FASN in GBM, FASN enzymes were found to be expressed at a higher level in glioma tissue than in normal neuronal tissue in both humans and rats (39). By using either RNAi-mediated knockdown of FASN or the FASNi cerulenin, this study showed an induction of apoptosis and an accumulation of cells in the S phase. A potential explanation for this phenomenon comes from Chen et al., who showed that palmitoylation of the transcription factor XBP1 protected glioma cells from ER stress–induced cell death (40). Inhibition using cerulenin (or the palmitoylation inhibitors 2-bromopalmitate and tunicamycin) arrests cells in the G_2_ phase and induces apoptosis, which synergizes with temozolomide to kill glioma cells both in vitro and in vivo. Interestingly, another group identified that the expression of FASN could also be used as a peripheral biomarker for GBM (41). In this study, the authors found that FASN levels are enriched in extracellular vesicles derived from GBM cell lines and human plasma. The findings of this work not only support studies showing the increase in FASN levels in GBM tissues but also suggest that plasma extracellular vesicle levels can be used as a noninvasive biomarker for GBM.

As FAS promotes multiple aspects of GBM metabolism, it is likely that the FA composition of the diet can influence GBM growth. Silver et al. recently found that consumption of a high-fat diet influences sulfur metabolism in murine models of GBM (42). The consumption of a high-fat diet caused FA accumulation in tumors through processes that resulted in increased glioma stem cell populations, promotion of tumor growth, and protection from necrotic cell death. This preference comports with another study demonstrating that glioma stem cells have increased levels of the FA scavenger receptor CD36 on their surface (43). In this study, CD36 expression was upregulated on glioma stem cell populations, which promoted enhanced self-renewal capacities and enhanced tumor growth in vivo. Blocking CD36 by either pharmacologic or genetic inhibition resulted in reduced stem cell phenotypes and reduced tumor growth in vivo. Therefore, the exogenous uptake of FAs may also contribute to gliomagenesis by enhancing stem cell phenotypes.

#### Lipid droplets store excess lipids in a cell.

While both MUFAs and saturated FAs can be incorporated into lipid bilayers, triglycerides, or lipoproteins, MUFAs have a unique role in promoting the formation of lipid droplets (LDs). LDs are intracellular stores of lipid and cholesterol esters stored in specialized vacuoles that can be rapidly liberated and oxidized in the mitochondria to produce ATP (12). LDs contain both FA esters (as triacylglycerols) and cholesterol esters, which are regulated by the enzymes DGAT1/2 and ACAT1/2 (also called SOAT1/2), respectively. LDs in GBM correlate with the grade of malignancy, and negatively prognosticate survival (44). It was identified that SOAT1 was responsible for LD formation, and inhibition using shRNA or the SOAT inhibitor (SOATi) avasimibe blunted LD formation and SREBP-1–mediated FAS. In a similar study by the same group, inhibition of DGAT1 (by either shRNA or the DGAT1i A-922500) prevented LD formation (45), and induced cell death by excessive FAO-induced ROS. This study mimics previously published work in the context of breast cancer brain metastasis (BCBM) (46), in which FABP7 is required for LD formation, and shRNA inhibition leads to excessive mitochondrial FAO and LD depletion. In this study, FABP7 depletion prevented the establishment of BCBM in murine models of the disease, suggesting that LD formation is essential for tumor cell survival in the CNS. Another recent study highlights the preferential accumulation of LDs in GBM organoid cores and in hypoxic/pseudopalisading regions of GBM (47). Supporting these observations, the expression of a critical LD-forming enzyme, hypoxia-inducible lipid droplet–associated (HILPDA), was also found to be correlated to these same hypoxic/pseudopalisading regions. Considering this work, and the biology of LDs described above, it appears that LDs might enable GBM cells to survive under hypoxia/nutrient-limiting conditions by acting as a reservoir for FAs and cholesterol. Indeed, research has demonstrated that, while yeast cells unable to produce LDs do not have perturbation in growth under steady-state conditions (48), LDs are essential in maintaining cellular survival under nutrient deprivation (49). Lastly, there is substantial literature suggesting that the LD formation both is antiapoptotic and promotes resistance to chemotherapies (reviewed extensively in ref. 50). The results of these studies demonstrate that LD homeostasis is critical to the maintenance of GBM growth and may be a valuable therapeutic target.

#### A metabolic switch between anabolism and catabolism.

**Figure 1:**
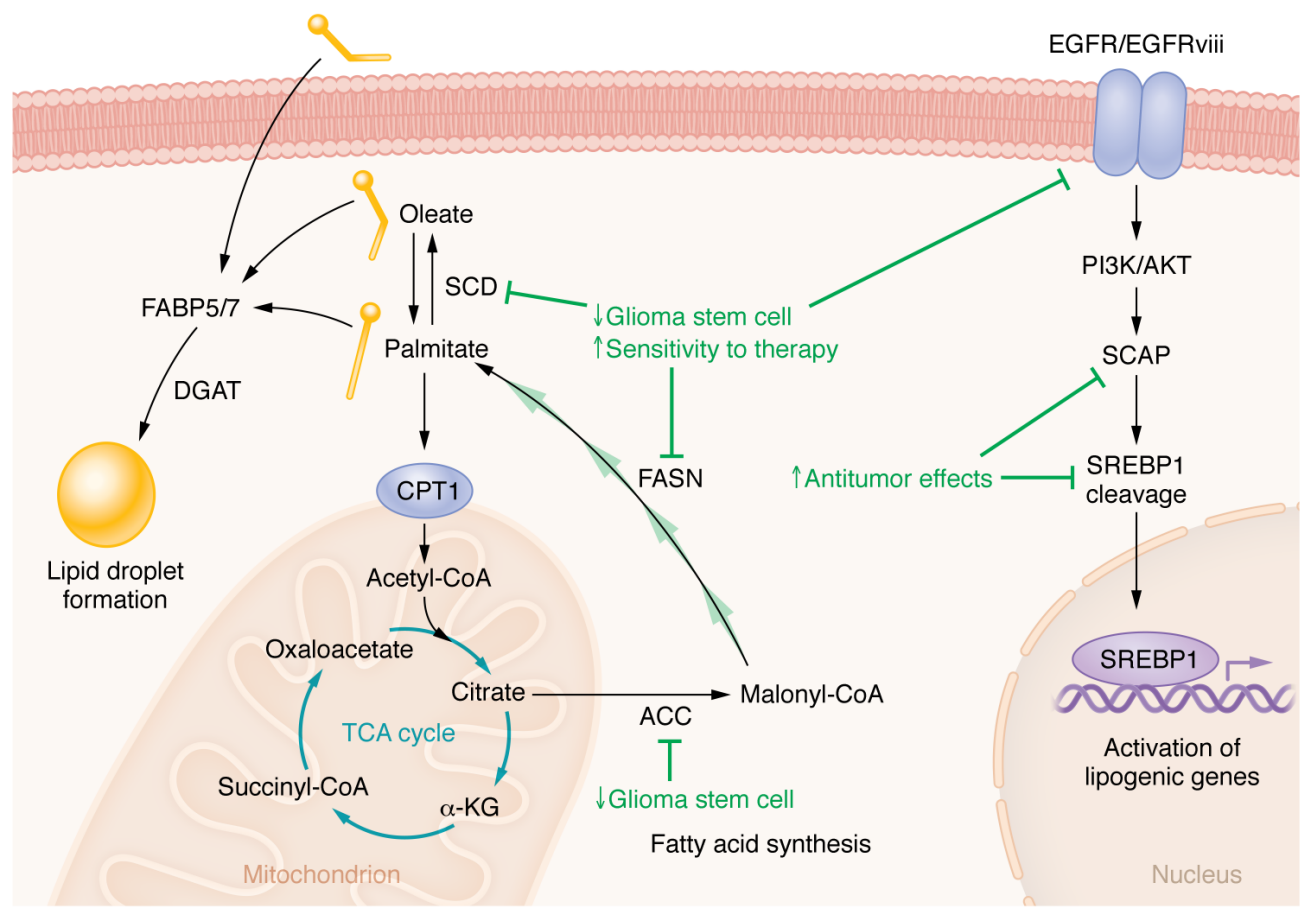
Perturbing FA anabolism in GBM. FA anabolic processes are critical for cellular growth, and blockade of anabolism is mostly associated with inhibition of glioma stem cell phenotypes and sensitization to anti-GBM therapies (anabolism-targeting strategies and their effects are shown in green). A master regulator of lipid biosynthesis in cells is EGFR-mediated SREBP-1 activation, and several studies have demonstrated that perturbation of this axis has strong antitumor properties. At the nexus of anabolism and catabolism is stearoyl-CoA desaturase (SCD), and there have been considerable efforts to inhibit these enzymes in both GBM and other tumors due to the tumor’s dependence on them to control FA desaturation. Blockade of SCD exerts powerful anti-GBM effects both in vitro and in vivo. FA synthesis is governed by both the multifunctional enzyme fatty acid synthase (FASN) and acetyl-CoA carboxylases (ACC1/2) . Targeting these enzymes prevent Glioma stem cell phenotypes and can promote other antitumor therapies. The role of each of these processes in tumors, their spatiotemporal location and activities, and how they promote therapy resistance are under active investigation.

The mechanism controlling the saturation of a lipid between a MUFA and a saturated FA is an essential metabolic process in cells and has been broadly implicated as a switch between anabolic and catabolic processes in tumors (51). The reversible process controlling MUFA–saturated FA interconversion is catalyzed by a series of enzymes called stearoyl-CoA desaturases (SCD1–SCD4 in mice, SCD1 and SCD5 in humans; ref. 52), which are responsible for the conversion of stearoyl-CoA (saturated FA) to oleoyl-CoA (MUFA). This enzyme family is the rate-limiting step of this process and the only way in which to make de novo MUFAs; otherwise, MUFAs must be obtained via dietary intake (53). Previous work has demonstrated that the SCD interconversion pathway is essential for chemoresistance (54) and, highlighting its role in anabolism, is essential in maintaining cell membrane stability in GBM (55). Indeed, the Badr laboratory recently identified that SCD activities are essential for GBM growth (56). In this study, shRNA targeting SCD or treatment with an SCDi abrogated glioma growth in vivo and dramatically inhibited stem cell frequency in limiting dilution assays. This observation and others, combined with the central role of SCD across multiple types of cancer, have motivated extensive work to identify specific inhibitors of SCD in cancer (57). However, in spite of the immense efforts to develop SCDi, SCD activity in GBM can be retained during SCDi therapy through a FOSB-mediated evasion mechanism (58). The heterogeneity of GBM can also be observed in the context of these enzymes. A recent study highlighted the expression of SCD/FADS2 changes depending on intratumoral location, with the lowest expression near the necrotic cores of freshly excised human GBM tissue (59), which should be considered when this axis is targeted for GBM therapy. While none of these compounds are in the clinic yet for GBM therapy, it remains an attractive option for therapeutic targeting. An overview of FAS in the regulation and growth of GBM can be found in Figure 1.

### FA catabolism in GBM

Two types of FAO occur in mammalian cells: α-oxidation and β-oxidation. α-Oxidation is a unique process that occurs in specialized vacuoles termed peroxisomes, and its role is to remove a single carbon from the carboxy terminus of certain lipids, allowing for their subsequent β-oxidation (60). Peroxisomes are also the site for the β-oxidation of lipid species known as very-long-chain fatty acids (VLCFAs). VLCFAs are FAs that are longer than 22 carbons; they are in fact so long that they cannot be metabolized in the mitochondria and must be catabolized in peroxisomes (61). Despite a recent study demonstrating that a specific molecular subtype of GBM (proneural) has an enrichment in these VLCFA species (62), only two publications have directly examined peroxisomes in GBM (63, 64). More research clearly needs to be done on these vacuoles and FA species in GBM.

Most of the research on FAO regards β-oxidation within the mitochondria. It is critical to note that only saturated carbon chains can be broken down via the process of FAO. Unsaturated FAs require several additional enzymatic steps that shift and saturate double bonds before being completely oxidized (65). Saturated FAs are required for FAO because trafficking of FAs is principally controlled by the carnitine-palmitate shuttle (CPT1), which only accepts saturated FAs (66). In the context of GBM, several studies have indicated that FAO is a relevant pathway for tumor growth and potentially a target for therapeutic intervention (29, 67–70). An influential study on this topic found that many of the enzymes required for FAO are abundant in GBM, and that glioma cells can readily oxidize lipids that promote cellular proliferation (67). Furthermore, the administration of etomoxir via an osmotic pump extended animal survival in murine models of GBM (67). Mechanistic insights into the regulation of FAO in GBM came from recent work demonstrating that palmitate oxidation depended on the expression of acyl-CoA–binding protein (ACBP), and this enzyme is elevated in brain tumor tissue compared with healthy brain. Subsequent knockout of ACBP stymied tumor growth in multiple GBM models in vitro and in vivo (70).

Recent work demonstrated that the upregulation of FAO occurs in niches in which nutrient deprivation is predominant and that cellular proliferation in these conditions is inhibited by etomoxir treatment (69). The authors found that dual FAO inhibition (using etomoxir) and inhibition of glucose metabolism (using 2-Deoxy-D-glucose [2-DG]) yielded a small but significant benefit in animal models of GBM. These data comport with another recent study that demonstrates that autophagy and subsequent FA catabolism facilitate glioma growth under mTORC1 activation (71). In another recent study, the authors performed an RNAi screen on glioma stem cells and determined that the mitochondrial enzyme medium-chain acyl-CoA dehydrogenase (MCAD) was critical for GBM growth (72). When MCAD was inhibited in GBM cells (either genetically of pharmacologically), it caused accumulation of medium-chain FAs, lipid peroxidation, and mitochondrial damage leading to apoptosis. This study demonstrates that the breakdown of medium-chain FAs is critical in preventing lipid peroxidation–induced cell death in stem cell populations.

Another interesting use of FAO by GBM cells is to evade antitumor responses elicited by radiation therapy (29). Interestingly, radiation upregulates FAO, generating citrate, which provides the substrate to acetylate RelA, thus promoting CD47 expression. This upregulation prevents phagocytosis by macrophages after radiotherapy, leading to tumor regrowth. Using both etomoxir and CRISPR-mediated KO of FAO enzymes, FAO inhibition could synergize with CD47 blockade as a potential therapy for GBM (29). The sum of these studies supports the hypothesis that the catabolism of FAs via FAO is a process relegated to cells undergoing stress and not those under steady-state proliferation.

Ketone body metabolism is another by-product of FAO that is actively being explored in the GBM space. Typically, ketone bodies are generated as a by-product of FAO, in which two molecules of acetyl-CoA generated from FAO are connected to form acetoacetate, a precursor to the ketone β-hydroxybutyrate (or the waste product acetone) (73). These ketone bodies can be shuttled out of the cell, and then can be reimported into cells to provide acetyl-CoA for use in the TCA cycle. Importantly, ketone bodies can enter the blood-brain barrier (BBB) via endothelial monocarboxylate transporters (MCT) (74) and, under conditions of prolonged fasting, can even replace glucose as the main energy source for the brain (73, 75, 76). A recent study supports that this may occur in GBM by showing that a ketogenic diet may promote tumor growth (77). However, the role of ketones in GBM is complicated by the fact that previous work suggested that GBM cells were not able to metabolize ketones as well as normal neuronal tissue (78). In fact, this study was used a rationale for a clinical trial using a ketogenic diet for GBM (79). While no toxicities were observed, the role of ketogenesis in GBM progression is still unclear.

**Figure 2:**
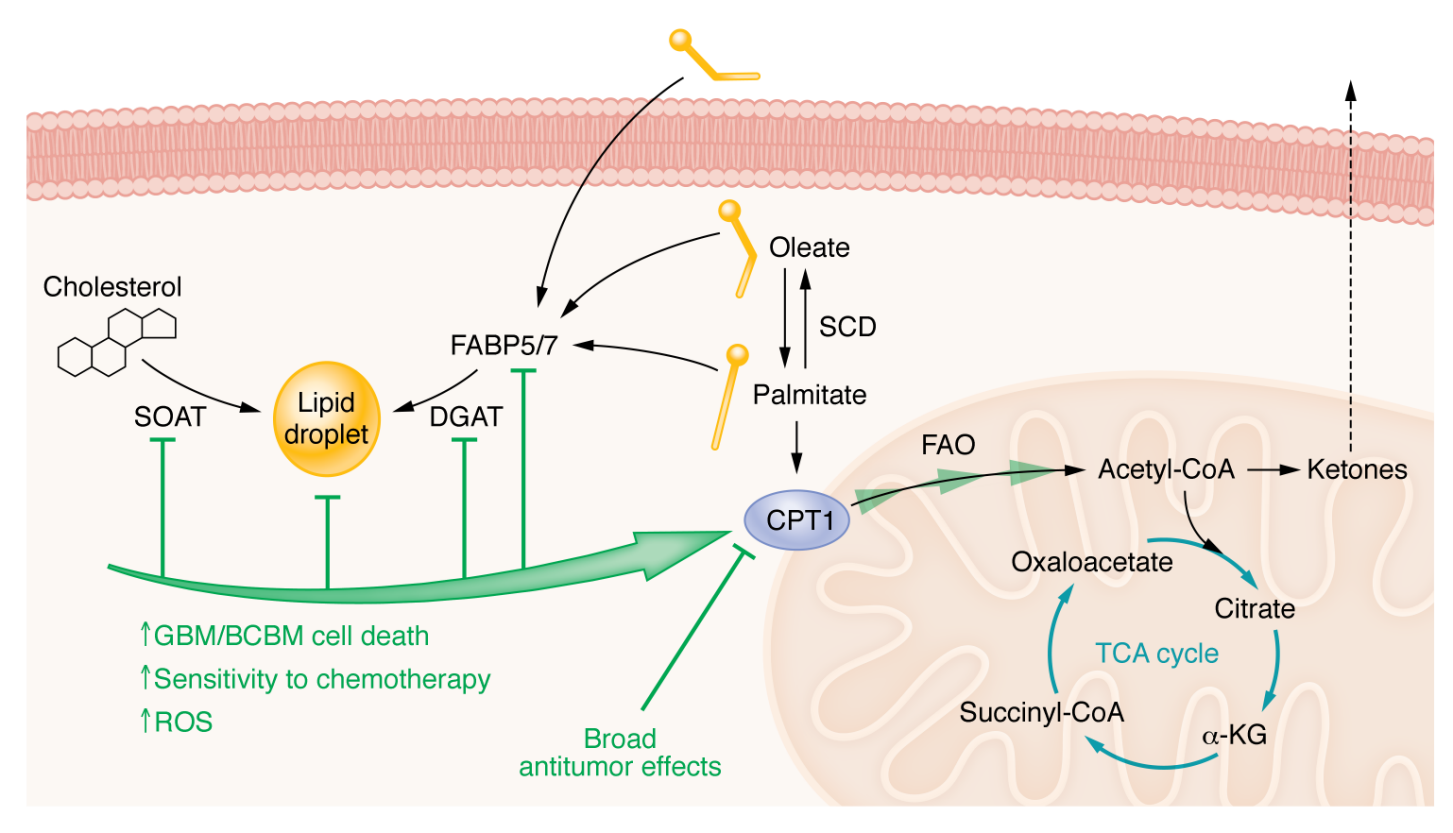
Perturbing FA catabolism in GBM. FA catabolism is the process by which lipids are broken down to generate energy and other metabolic intermediates for cells. This is a tightly controlled process, as too much FA oxidation (FAO) can be toxic to cells. For example, in lipid-rich environments glioma cells have a mechanism by which they store excess FAs/cholesterol in lipid droplets. Under conditions of bioenergetic stress, FAs are liberated from these droplets to provide a robust source of ATP via FAO. The blockade of lipid droplet formation using strategies that targeted various steps (green) caused erroneous mitochondrial FAO resulting in accumulation of ROS and cell death in brain tumors. The bringing of FAs into the mitochondria is controlled by the essential enzyme CPT1 (the CPT1A isoform is rate limiting). Many studies have used genetic and pharmacologic inhibition of CPT1A and have shown broad antitumor activities. Ketone bodies are both a by-product of FAO and a way to transport energy in an intercellular fashion. The protective or deleterious role of ketones in brain tumors is still not clear and is a hotly debated topic in the field of FA metabolism.

While these studies highlight the importance of FAO in stress adaptation by GBM, it is critical to understand that a useful inhibitor has yet to reach the clinic. Etomoxir, which was used in almost every study described above, was initially discovered as an FAOi that blocks the actions of CPT1A (80). However, studies have since shown that at commonly used doses, etomoxir inhibits mitochondrial oxidative phosphorylation (81), inhibits Tregs and memory T cells (81), and induces severe oxidative stress (82) independent of CPT1A-mediated FAO. Partial FAOi (pFOXi) such as ranolazine have also been explored (83) and may indeed exert some antitumor effects in GBM (71, 84), although the antitumor effects are minimal. Another pFOX, trimetazidine, has been minimally explored in cancer (85) and may be another useful tool in targeting FAO in GBM. In summation, although FAO may be a relevant therapeutic target for GBM, especially for cells undergoing metabolic or therapeutic stress, there is not currently a useful way to target this pathway in the clinic. An overview of FAO in GBM therapy can be found in Figure 2.

#### FAO and immune suppression.

While the above section focuses on the direct role of FAO in brain tumors, a substantial body of work has demonstrated that FAO contributes to various aspects of immunity, particularly regarding immune suppression. The first work to indicate that FA metabolism is relevant to immunosuppression demonstrated that treatment with etomoxir inhibited myeloid-derived suppressor cell metabolism and immunosuppressive functions in tumors (though see above for limitations of this approach) (86). In this study, the authors demonstrated that etomoxir or the pFOX ranolazine restricted the CD4^+^ and CD8^+^ T cell–dependent growth of MC38 tumors in vivo. Moreover, inhibition of FAO worked in concert with adoptive cell therapy, suggesting that FAO is part of an immunosuppressive axis in cancer. In the context of GBM, we previously found that Tregs have elevated levels of multiple FA transporters compared with other T cell subsets specifically within the GBM environment (87). Furthermore, inhibition of FA transport or FAO inhibition (via etomoxir) reduced Treg-mediated immunosuppression and resulted in significant survival benefits in vivo (which were also dependent on adaptive immunity). Subsequent studies have validated this observation, some showing that expression of the FA transporter CD36 is essential for Treg function in tumors (88).

### Control of ferroptosis by PUFAs and MUFAs in GBM

The third and most recent discovery in the field of FA metabolism is the non-apoptotic cell death termed ferroptosis (89). In this unique form of cell death, iron inside the cell reacts with (mainly) mitochondrially derived hydrogen peroxide in a process termed the Fenton reaction. The product of these reactions is hydroxyl radicals, which then react with PUFAs at lipid bilayers to form lipid hydroperoxide (LOOH). These highly reactive compounds are the effectors of ferroptosis and are negatively regulated by ROS scavenging systems, most notably the glutathione-dependent GPX4 and FSP1 system (an extensive review of the basics of ferroptosis can be found in ref. 90). During the past 10 years of research into ferroptosis, it has become clear that cancer has a unique relationship with this pathway. It has been well established that tumors rely on iron uptake for growth as compared with nonmalignant cells (91), generate substantial ROS (92), and contain large amounts of PUFAs (93–95), and thus tumors are thought to be uniquely susceptible to inducers of ferroptosis. However, the systems responsible for resolving lipid ROS (glutathione peroxidase/catalase/peroxiredoxins, etc.) are also concordantly increased in tumors, endowing them with the ability to handle the increased oxidative stress (an extensive review on ferroptosis in cancer can be found in ref. 96). However, this ability also may put tumors at a unique disadvantage, in that perturbation of their ferroptotic resolving pathway might sensitize them to chemotherapies. Indeed, much work has indicated that modulation of ferroptosis can lead to durable antitumor responses and can even prevent resistance to other typical chemotherapies (97).

**Figure 3:**
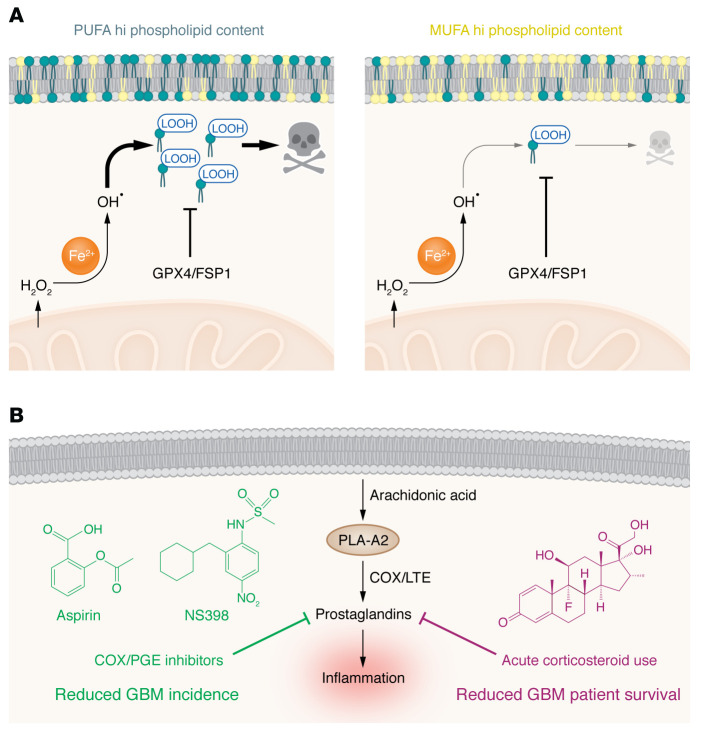
Ferroptosis and lipid signaling in GBM. (**A**) Ferroptosis is a recently discovered form of non-apoptotic cell death that is dependent on polyunsaturated FAs (PUFAs) that are oxidized by reactive oxygen species (ROS) and iron to generate lipid hydroperoxides (LOOH). Ferroptosis is avoided by tumors via the lipid ROS–resolving enzymes glutathione peroxidase 4 (GPX4) and ferroptosis suppressor protein 1 (FSP1). Ferroptosis is also inhibited by exogenous administration of monounsaturated FAs (MUFAs), demonstrating that the PUFA/MUFA balance may be predictive of ferroptosis potential in tumor cells. The role of these processes in GBM, and whether they can be perturbed to enhance anti-GBM therapies, are largely unknown. (**B**) Specific PUFAs, particularly arachidonic acid (AA) generated by the actions of phospholipase A_2_ (PLA-A2), are used to generate the potent inflammatory compounds collectively called prostanoids. Of these, prostaglandins are the most well studied, and long-term use of inhibitors of prostaglandin generation, such as the COX/PGE inhibitors aspirin and NS398, are negatively associated with GBM occurrence in numerous studies. However, the clinical use of steroids such as dexamethasone, which also inhibits prostaglandins (and numerous other processes), is associated with worse outcomes in GBM and resistance to immunotherapies for the disease. The results of these studies suggest that long-term blockade of inflammation may prevent GBM incidence. However, once malignancy is identified, acute blockade of inflammation inhibits patient survival and immunotherapeutic efficacy. The mechanisms behind these diverging phenomena are still being investigated.

PUFA biology is at the very center of this process. Indeed, the expression of the PUFA catabolic enzyme ACSL4 is required for ferroptosis to occur (98). This enzyme is required for incorporation of the PUFA arachidonic acid (AA) into the phospholipid bilayer and demonstrates that the incorporation of PUFAs into phospholipids is an essential component of ferroptosis. Supporting the role of PUFAs in this process, the availability of PUFAs has been used as a direct indicator of a sensitivity of gastric cancer cells to ferroptosis (94); in another fascinating study, the authors found that supplementation of exogenous MUFAs to fibrosarcoma cells led to a ferroptosis-resistant state (99). Therefore, the abundance of PUFAs is directly linked to sensitivity to therapeutic intervention. The literature is inconsistent regarding the abundance and biology of PUFAs in GBM, although historical studies have indicated that their levels are increased compared with those in normal brain tissue (100–102). An overview of ferroptosis and its regulation can be found in Figure 3A.

### The prostaglandin paradox in GBM

Another consideration regarding the role of FAs in GBM pathology is their conversion to a group of compounds collectively termed prostanoids. In these studies, membrane phospholipids are cleaved principally by the enzyme phospholipase A_2_ (and to a lesser extent by phospholipase C/D members) to generate AA, then acted upon by several different enzymes to produce these signaling compounds (103). The most well studied of these is prostaglandin E_2_ (PGE_2_), which is made as a by-product of cyclooxygenase-2 (COX-2) (104). Extensive work on PGE_2_ has been explored in cancer, with the consensus observation that it promotes malignancy (105). In GBM, COX-2 expression is positively correlated with GBM grade and negatively prognosticates survival (106). Pharmacologic targeting of COX in GBM has been performed both in vitro and in vivo. Nonsteroidal antiinflammatory drugs are the most well-known inhibitors of COX enzymes and, owing to their widespread usage, have elicited numerous results on their role in GBM progression (107–113). Interestingly, a recent case-controlled meta-study found a significant (although marginal) reduction in glioma incidence in aspirin users (109), consistent with previous articles on the topic (111, 113). Treatment with the COX-2i NS-398 prevented proliferation, migration, and tumor spheroid generation of GBM cells in vitro (107). Another, more recent study also examined NS-398 and showed a similar reduction in tumor sphere growth rate, extracellular vesicle release, and increase in autophagy (108). The results of these studies suggest that prostaglandin production is overall a protumoral FA derivative.

However, confounding the issue of targeting of these AA-derived molecules, especially in the context of GBM, is that steroid use has been directly correlated with tumor progression. Patients with GBM frequently have severe intracranial inflammation, either before or after surgery, which has historically required the administration of dexamethasone (114). Dexamethasone acts as a switch to turn off AA synthesis and subsequent prostaglandin synthesis, inhibiting the prostanoid pathways (among manifold other effects in preventing inflammation). However, contrary to the data regarding long-term use of antiinflammatory drugs preventing GBM initiation, acute blockade of this pathway after diagnosis (and during initial surgery) has clearly deleterious effects on patient survival and antitumor immunity (115–119). Indeed, increasing amounts of dexamethasone directly inhibited the efficacy of a new transcriptionally regulated IL-12 oncolytic adenoviral therapy (115), responses to checkpoint immunotherapy (118), and other therapeutics not directly associated with immunity (120, 121). Therefore, while inhibiting AA metabolism may appear to be a useful strategy to target tumor initiation, its unwanted effects on adaptive immunity must be taken into consideration, especially once the tumor has been established. A simple schematic highlighting this apparent contradiction can be found in Figure 3B.

## Characterizing FA composition of brain tumors

An issue hindering a true understanding of the FA milieu of brain tumors is the complex heterogeneity that underlies the disease. Highlighting this complexity is a study that examined the use of desorption electrospray ionization mass spectrometry (DESI-MS) to spatially profile the lipid composition of 36 brain tumors of different histological grades (122). While use of this methodology revealed FA signatures that differed between histological grades, the differences were small, and no specific lipid profile could be observed in these tumors. A recent landmark study highlighting the complexity of FAs in brain tumors examined the lipidome of 99 GBMs (62). In this work, the authors analyzed 582 lipid species from 75 tumors, compared them against seven normal brain tissues, and found significant differences in more than 500 of the lipid species. In keeping with the heterogeneity of these tumors, the authors found that the lipidome of brain tumors differs based on Isocitrate dehydrogenase (IDH) status and tumor molecular subtype, which are unique in comparison with normal brain tissues. For example, the authors found that the mesenchymal subtype of GBM had an increase in overall levels of glycerolipids (i.e., triacylglycerols) and a decrease in glycerophospholipids, whereas in the proneural subtype the authors found enrichment in VLCFAs and glycerophospholipids with PUFA side chains. Again, these changes were relatively small, and with the identification of hundreds of unique lipid species, many with unknown biological functions, this study brings up more questions while providing few answers about FA metabolism in GBM.

One critical aspect to consider when assessing publications on the lipidomics of brain tumors is the inherent heterogeneity of these tumors. Typically, for quantification based on liquid chromatography–mass spectrometry, researchers are generally limited to assessing by the small pieces of tumor tissue. This is problematic since FA metabolism changes depending on intratumor location (47, 59, 122). Therefore, care needs to be taken not to overinterpret any data coming from whole GBM tissue. Another technical aspect that is critical to assess is that in vitro or ex vivo measurement of FAs may not reflect the reality in vivo. Besides the typical limitations of using cell lines, many studies that use patient-derived xenografts describe GBM by the molecular subtypes initially described in ref. 123. However, the reality is that within a brain tumor, all molecular subtypes exist (124), so researchers should be cautious in associating a certain FA metabolic phenotype with GBM molecular subtypes. The third technical issue to assess is the methodology of acquisition and the inherent limitations of the associated techniques. Many (mainly) mass spectrometric techniques are used for lipidomics, and each comes with unique advantages and disadvantages. A detailed review of lipidomic methodologies and good descriptions of technical issues can be found in refs. 125–128.

## Conclusions and contextualization

FA metabolism, though the focus of this Review, is only a small part of the metabolic machinery inside cells. Furthermore, metabolism is inherently plastic and can rapidly change depending on nutrient availability. A great example comes from a study that identified metabolic plasticity in GBM cells with stem cell/slow-cycling phenotypes (129). This work showed that “slow-cycling” stem cells can upregulate FA transport when glucose transport is inhibited and, conversely, upregulate glucose transport when FA metabolism is inhibited. Future studies need to examine how tumors rewire their metabolism when FA metabolism is targeted to promote antitumor responses.

This Review demonstrates that FA metabolism is a central component of brain tumor biology and shows the strategies to perturb these pathways. However, a unique challenge to testing of these strategies in humans is the requirement that these compounds must be able to cross the BBB. Table 1 summarizes both the compounds used to target FA metabolism in brain tumors and their potential ability to cross the BBB. Despite the tremendous efforts in researching FA metabolism in GBM, we still understand very little about the mechanistic biology of these molecules. Part of the confusion and challenge is the sheer volume of lipid species identified by newer lipidomic technologies. Do these lipid species play a specific role in membrane stability, and are they used for a currently unknown biological process? Are there context-dependent or spatial differences in FA metabolism that have not been accurately captured in studies to date? Another limitation on our understanding of FA metabolism in GBM is that the measurement of lipids comes from lysed tumor tissues and does not necessarily reflect the actual availability of FAs to cells within the CNS. In a recent paper, a well-established method of interstitial fluid isolation (130) determined the metabolome of the CNS to assess nutrient availability for potentially metastasizing cells (131) and found a minimal availability of FAs in the interstitium of the brain. Subsequent experiments demonstrated that brain-metastatic cells require the FASN complex to metastasize to the brain. Thus, in the future we must expand our efforts, techniques, and scope of research to truly understand FA metabolism in GBM.

## Figures and Tables

**Table 1:**
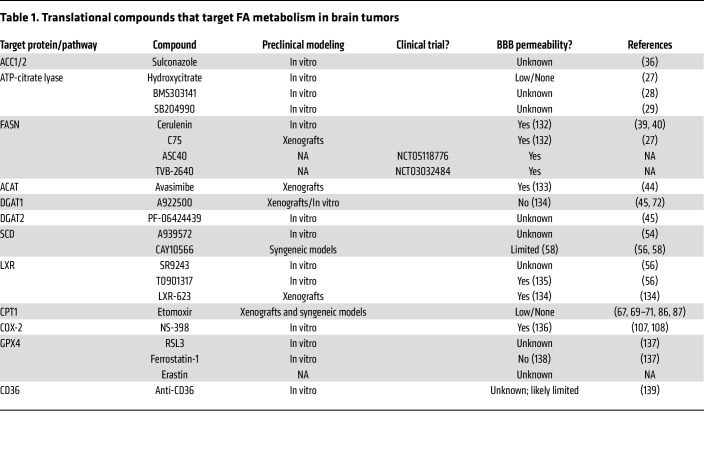
Translational compounds that target FA metabolism in brain tumors

## Version 1 01/03/2023

Electronic publication
